# Generalized Magnetic Field Effects in Burgers' Nanofluid Model

**DOI:** 10.1371/journal.pone.0168923

**Published:** 2017-01-03

**Authors:** M. M. Rashidi, Z. Yang, Muhammad Awais, Maria Nawaz, Tasawar Hayat

**Affiliations:** 1 Shanghai Key Lab of Vehicle Aerodynamics and Vehicle Thermal Management Systems, Tongji University, Jiading, Shanghai, China; 2 Department of Mathematics, COMSATS Institute of Information Technology, Attock, Pakistan; 3 Department of Mathematics, Quaid-i-Azam University, Islamabad, Pakistan; The Ohio State University, UNITED STATES

## Abstract

Analysis has been conducted to present the generalized magnetic field effects on the flow of a Burgers' nanofluid over an inclined wall. Mathematical modelling for hydro-magnetics reveals that the term “$\sigma B_{0}^{2}u/\rho$” is for the Newtonian model whereas the generalized magnetic field term (as mentioned in Eq 4) is for the Burgers’ model which is incorporated in the current analysis to get the real insight of the problem for hydro-magnetics. Brownian motion and thermophoresis phenomenon are presented to analyze the nanofluidics for the non-Newtonian fluid. Mathematical analysis is completed in the presence of non-uniform heat generation/absorption. The constructed set of partial differential system is converted into coupled nonlinear ordinary differential system by employing the suitable transformations. Homotopy approach is employed to construct the analytical solutions which are shown graphically for sundr5y parameters including Deborah numbers, magnetic field, thermophoresis, Brownian motion and non-uniform heat generation/absorption. A comparative study is also presented showing the comparison of present results with an already published data.

## Introduction

Analysis of non-Newtonian fluids is an active area of research for the last few years. Many industrial fluids including certain oils, shampoos, paints, cosmetic products, polymers, colloidal fluids, suspension fluids, ice cream etc. are characterize as the non-Newtonian fluids. The diverse rheological properties of the non-Newtonian fluids results into division namely the rate type (includes the Maxwell model, Jeffery model, Oldroyd model and the Burgers model etc), the differential type (includes the second grade model, third grade model etc) and the integral type (includes Sisko model etc.). The Burgers' fluid model has been proposed to predict the properties of relaxation and retardation effects simultaneously. Due to the complexities in terms mathematical modelling of the Burgers’ fluid model, very little has been reported for the Burgers’ model. For-instance Jamil and Fetecau [1] computed some exact solutions for rotating flows of a generalized Burgers’ fluid in cylindrical domains. Authors have considered the fluid flow between two co-axial cylinders and employed the Laplace and Hankel transforms for the solutions procedure. A note on the longitudinal oscillations of a generalized Burgers fluid in cylindrical domains has been presented by Fetecau et al. [2]. Authors have presented the effects of longitudinal oscillations and established Fourier-Bessel series. An exact solution of start-up flow for the ractional generalized Burgers’ fluid in a porous half space is presented by Xue et al. [3]. They have utilized the modified Darcy’s law for the mathematical modelling in a porous medium and employed Fourier sine and fractional Laplace transform to compute the exact solution of the velocity distribution. Khan et al. [4] computed the exact solutions for some oscillating motions of a fractional Burgers’ fluid. Authors have incorporated fractional calculus approach in the constitutive relationship model. The expressions for the velocity field and the resulting shear stress that have been presented. Similar solutions for generalized Oldroyd-B, Maxwell and second grade fluids can be computed as a limiting cases. Hydromagnetic flow and heat transfer of a generalized Burgers’ fluid due to an exponentially accelerating plate with radiation effects has been analyzed by Liu et al. [5]. The authors have presented the flow over an exponentially accelerating wall. Exact solutions for the velocity and temperature field are presented in integral and series form in terms of the GG functions. Maryam et al. [6] presented the peristaltic flow of Burgers’ fluid with complaint walls and heat transfer. Authors have studied a theoretical analysis to investigate the peristaltic flow and heat transfer characteristics of Burgers’ fluid in a channel governed by the propagation of sinusoidal waves. Various plots are presented to discuss the considered rheology. Awais et al. [7] investigated the heat transfer in flow of Burgers’ fluid during melting process. The two-dimensional flow equations are modeled and then simplified by employing boundary layer analysis. The solution to the arising nonlinear problem is computed and interpretation of various emerging parameters is given through graphs.

In the recent years, particles of nanometre-size (normally less than 100 nm proposed by Choi [8]) termed as nanofluid are utilized for dispersing in base liquids in order to enhance the thermal conductivity even at low solid concentrations. They are also very stable and have no additional problems such as erosion, additional pressure drop and the low volume fraction etc. The enhanced thermal behavior of nanofluids could provide a basis for an enormous innovation for heat transfer intensification, which is major importance to a number of industrial sectors including transportation, nuclear reactors, electronics as well as biomedicine and food. In view of these fact various recent researchers have made several investigation to study the nanofluid rheology in Newtonian and non-Newtonian fluids. For-instance Khan and Pop [9] presented the boundary-layer flow of a nanofluid past a stretching sheet. Authors have presented a similarity solution is presented which depends on the Prandtl number, Lewis number, Brownian motion number and thermophoresis number. Makinde and Aziz [10] studied the boundary layer flow of a nanofluid past a stretching sheet with a convective boundary conditions. They have extended the work of Ref. [9] for convective boundaries which are considered to be more generalized boundary conditions. Numerical study of the flow and heat transfer of a nanofluid over a stretching sheet has been analyzed by Rana and Bhargava [11]. They have analyzed the flow over a nonlinearly stretching surface and solved the considered problem using finite element method (FEM) with a local non-similar transformation. Hamad and Ferdows [12] presented the similarity solution of boundary layer stagnation-point flow towards a heated porous stretching sheet saturated with a nanofluid with heat absorption/generation and suction/blowing. They have utilized the Lie group theory to analyze the outcomes of the problem. Heat generation/absorption effects in a boundary layer stretched flow of Maxwell nanofluid has been analyzed by Awais et al. [13]. Authors have presented analytic and numeric solutions and presented several plots and numerical results to give the exact insight of the considered problem.

The purpose of the present analysis is to investigate further in the direction of nanofluids’ rheology. Therefore we have studied the Burgers’ nanofluid flow. Generalized magnetic field terms is presented for the over an inclined wall with non-uniform heat generation/absorption and gravitational effects. Mathematical modelling has been performed in the presence of applied magnetic field. It is noted that magnetic field term (which appear in the momentum equation) for rate type fluids is quite different then of differential type fluids. The governing nonlinear partial differential system is converted into system of coupled ordinary differential equations using appropriate transformations. The resulting equations are solved analytically by using HAM [14–21] and the obtained results are presented graphically. A comparative study with an already published data is presented showing the nice agreement in derived results. Some new published papers can be found in [22–24].

## Mathematical Formulation

We considered the boundary layer flow of an incompressible Burgers' nanofluid over an inclined wall (makes an angle α with the vertical direction). Heat and mass transfer effects are considered combined with non-uniform internal heat generation/absorption phenomenon. Magnetic field **B** = {0,*B*_0_,0} is applied along the transverse direction. A conducting wall undergoes stretching phenomenon with velocity *U*_*s*_(*x*) = *cx*, where *c* is a positive dimensional constant. The fundamental laws representing the mass and momentum conservation yield

divV=0, (1)

$$\rho \frac{DV}{Dt}=-\nabla p+div\;S,$$ (2)

where an extra stress tensor in Burgers' fluid model satisfy

$$S+\lambda_{1}\frac{DS}{Dt}+\lambda_{2}\frac{D^{2}S}{Dt^{2}}=\mu (A_{1}+\lambda_{3}\frac{DA_{1}}{Dt}).$$ (3)

In above equations (*λ*_1_, *λ*_2_) are the relaxation effects and *λ*_3_ is the retardation effect. It is pointed out here that for *λ*_2_ = 0, the results for an Oldroyd-B fluid model can be deduced and for *λ*_2_ = *λ*_3_ = 0, one can achieve the results for an Maxwell fluid model. Moreover the results for the Newtonian fluid model can be obtained by setting *λ*_1_ = *λ*_2_ = *λ*_3_ = 0. Making use of Eq 3 in Eq 2 we get

$$\begin{array}{l} u\frac{\partial u}{\partial x}+v\frac{\partial u}{\partial y}+\lambda_{1}\left(\begin{array}{l} u^{2}\frac{\partial^{2}u}{\partial x^{2}}+v^{2}\frac{\partial^{2}u}{\partial y^{2}}\\ +2uv\frac{\partial^{2}u}{\partial x\partial y} \end{array}\right)+\lambda_{2}\left(\begin{array}{c} u^{3}\frac{\partial^{3}u}{\partial x^{3}}+v^{3}\frac{\partial^{3}u}{\partial y^{3}}+u^{2}\left(\frac{\partial^{2}u}{\partial x^{2}}\frac{\partial u}{\partial x}-\frac{\partial u}{\partial y}\frac{\partial^{2}v}{\partial x^{2}}+2\frac{\partial v}{\partial x}\frac{\partial^{2}u}{\partial x\partial y}\right)\\ +3v^{2}\left(\frac{\partial v}{\partial y}\frac{\partial^{2}u}{\partial y^{2}}+\frac{\partial u}{\partial y}\frac{\partial^{2}u}{\partial x\partial y}\right)+3uv\left(u\frac{\partial^{3}u}{\partial x^{2}\partial y}+v\frac{\partial^{3}u}{\partial x\partial y^{2}}\right)\\ +2uv\left(\frac{\partial u}{\partial y}\frac{\partial^{2}u}{\partial x^{2}}+\frac{\partial v}{\partial x}\frac{\partial^{2}u}{\partial y^{2}}+\frac{\partial v}{\partial y}\frac{\partial^{2}u}{\partial x\partial y}-\frac{\partial u}{\partial y}\frac{\partial^{2}v}{\partial x\partial y}\right) \end{array}\right)=\\ \nu \left\{\frac{\partial^{2}u}{\partial y^{2}}+\lambda_{3}\left(u\frac{\partial^{3}u}{\partial x\partial y^{2}}+v\frac{\partial^{3}u}{\partial y^{3}}-\frac{\partial u}{\partial x}\frac{\partial^{2}u}{\partial y^{2}}-\frac{\partial u}{\partial y}\frac{\partial^{2}v}{\partial y^{2}}\right)\right\}+g_{0}\beta_{T}\left(T-T_{\infty }\right)\cos\alpha \\ -\frac{\sigma B_{0}^{2}}{\rho }\left(u+\lambda_{1}v\frac{\partial u}{\partial y}+\lambda_{2}\left\{u\frac{\partial v}{\partial x}\frac{\partial u}{\partial y}-v\frac{\partial u}{\partial x}\frac{\partial u}{\partial y}+uv\frac{\partial^{2}u}{\partial x\partial y}+v^{2}\frac{\partial^{2}u}{\partial y^{2}}\right\}\right). \end{array}$$ (4)

Moreover the energy and mass fraction equations are

$$u\frac{\partial T}{\partial x}+v\frac{\partial T}{\partial y}=\alpha_{m}\frac{\partial^{2}T}{\partial y^{2}}+\tau \left\{D_{B}\frac{\partial C}{\partial y}\frac{\partial T}{\partial y}+\frac{D_{T}}{T_{\infty }}{\left(\frac{\partial T}{\partial y}\right)}^{2}\right\}+\frac{\bar{q}}{\rho c_{p}},$$ (5)

$$u\frac{\partial C}{\partial x}+v\frac{\partial C}{\partial y}=D_{B}\frac{\partial^{2}C}{\partial y^{2}}+\frac{D_{T}}{T_{\infty }}\frac{\partial^{2}T}{\partial y^{2}}.$$ (6)

Note that in above differential system *u* and *v* are the velocity components, *ρ* is the density of the fluid, *σ* is the electrical conductivity, *g*_0_ is the acceleration due to gravity, *α* is the inclination of the wall, *T* is the temperature of the fluid, *α*_*m*_ is the thermal diffusivity of ordinary fluid, *D*_*B*_ is the Brownian motion coefficient, *D*_*T*_ is the thermophoresis effect and $\bar{q}$ is the non-uniform heat generated $\left(\bar{q}>0\right)$ or absorbed $\left(\bar{q}<0\right)$ per unit volume respectively. The mathematical expression for non-uniform heat source/sink is given by

$$\bar{q}=\frac{ku_{s}}{x\nu }\left[A\left(T_{s}-T_{\infty }\right)f^{\prime }+B\left(T-T_{\infty }\right)\right],$$ (7)

whereas the wall stretching velocity *U*_*s*_ = *cx*, the wall surface temperature $T_{s}=T_{\infty }+T_{ref}\frac{cx^{2}}{2\nu }$ and the concentration at the wall $C_{s}=C_{\infty }+C_{ref}\frac{cx^{2}}{2\nu }$, where *T*_*ref*_ is the constant reference temperature and *C*_*ref*_ is the constant value of concentration at the wall respectively.

The associated wall conditions are given by

$$\begin{array}{l} u=U_{s},\;v=0,\;T=T_{s},\;C=C_{s},\;\text{at}\;y=0,\\ u=0,\;v=0,\;T\to T_{\infty },\;C\to C_{\infty },\;\text{as}\;y\to \infty . \end{array}$$ (8)

Making use of the following transformations

$$\eta =\sqrt{\frac{c}{\nu }}y,\;u=cxf^{\prime }(\eta ),\;v=-\sqrt{c\nu }f(\eta ),\;\theta (\eta )=\frac{T-T_{\infty }}{T_{s}-T_{\infty }},\;\phi (\eta )=\frac{C-C_{\infty }}{C_{s}-C_{\infty }},$$ (9)

Eq 1 is automatically satisfied and Eqs 4–6 yield

$$\begin{array}{l} f^{\prime \prime \prime }-f^{\prime 2}+ff^{\prime \prime }+\beta_{1}(2ff^{\prime }f^{\prime \prime }-f^{2}f^{\prime \prime \prime })+\beta_{2}(f^{3}f^{\prime \prime \prime \prime }-2ff^{{}^{\prime }2}f^{\prime \prime }-3f^{2}f^{\prime \prime 2})\\ +\beta_{3}(f^{\prime \prime 2}-ff^{\prime \prime \prime \prime })-M^{2}(f^{\prime }-\beta_{1}ff^{\prime \prime }+\beta_{2}f^{2}f^{\prime \prime \prime })+G\theta \cos\alpha =0, \end{array}$$ (10)

$$\theta^{\prime \prime }+\mathrm{Pr}\left(f\theta^{\prime }-2f^{\prime }\theta +N_{b}\phi^{\prime }\theta^{\prime }+N_{t}{\left(\theta^{\prime }\right)}^{2}\right)+Af^{\prime }+B\theta =0,$$ (11)

$$\varphi^{\prime \prime }+Sc\left(f\phi^{\prime }-2f^{\prime }\phi \right)+\frac{N_{t}}{N_{b}}\theta^{\prime \prime }=0,$$ (12)

along with the wall conditions

$$\begin{array}{l} f(\eta )=0,\;f^{\prime }(\eta )=1,\;\theta (\eta )=1,\;\phi (\eta )=1\;\text{at}\;\eta =0,\\ f^{\prime }(\eta )=0,\;\theta (\eta )=0,\phi (\eta )=0\;\text{as}\;\eta \to \infty , \end{array}$$ (13)

where *β*_1_, *β*_2_ and *β*_3_ are the Deborah numbers, Pr is the Prandtl number, *M* is the Hartman number, *N*_*b*_ is the Brownian motion phenomenon, *N*_*t*_ is the thermophoresis effect, *Sc* is the Schmidt number and *G* is the mixed convection parameter. It is noted that *G* > 0 corresponds to assisting flow whereas *G* < 0 represents opposing flow situation. For *G = 0* the results of forced convection phenomenon can be reproduced. Moreover *A* and *B* are the dimensionless coefficients of space and temperature dependent heat generation/absorption effects respectively. Note that for internal heat generation *A > 0* and *B > 0* whereas for internal heat absorption we have *A < 0* and *B < 0*. These quantities are defined as

β1=cλ1,β2=c2λ2,β3=cλ3,Sc=νDB,M2=σB02ρc,Nb=τDB(Cs−C∞)νG=GrxRex2,Grx=g0βT(Ts−T∞)x3ν2,Rex=Usxν,Nt=τDT(Ts−T∞)νT∞,Pr=ναm, (14)

The local Nusselt (*Nu*) and Sherwood (*Sh*) numbers have the following definitions

$$\begin{array}{l} Nu=\frac{xq_{s}}{k\left(T_{s}-T_{\infty }\right)},\;Sh=\frac{xj_{s}}{D_{B}\left(C_{s}-C_{\infty }\right)},\\ q_{s}=-k{\left(\frac{\partial T}{\partial y}\right)}_{y=0},\;j_{s}=-D_{B}{\left(\frac{\partial C}{\partial y}\right)}_{y=0} \end{array}$$ (15)

in which *q*_*s*_ and *j*_*s*_ represent the surface heat flux and surface mass flux respectively. In dimensionless form

$$Nu/Re_{x}^{1/2}=-\theta^{\prime }(0),\;Sh/Re_{x}^{1/2}=-\phi^{\prime }(0).$$ (16)

## Solution Methodology

In order to proceed for the solution we select the initial guesses for the velocity, temperature and mass fraction fields given by

$$f_{0}(\eta )=1-\exp(-\eta ),\;\theta_{0}(\eta )=\exp(-\eta ),\;\phi_{0}(\eta )=\exp(-\eta ),$$ (17)

and the auxiliary linear operators as

$$L_{f}=\frac{d^{3}f}{d\eta^{3}}-\frac{df}{d\eta },\;L_{\theta }=\frac{d^{2}\theta }{d\eta^{2}}-\theta ,\;L_{\phi }=\frac{d^{2}\phi }{d\eta^{2}}-\phi ,$$ (18)

The zeroth order deformation problems are constructed by the following expressions

$$(1-p)L_{f}[\hat{f}(\eta ,p)-f_{0}(\eta )]=p\hbar_{f}N_{f}\left[\hat{f}(\eta ,p),\hat{\theta }(\eta ,p)\right],$$ (19)

$$(1-p)L_{\theta }[\hat{\theta }(\eta ,p)-\theta_{0}(\eta )]=p\hbar_{\theta }N_{\theta }\left[\hat{f}(\eta ,p),\hat{\varphi }\left(\eta ,p\right),\hat{\theta }(\eta ,p)\right],$$ (20)

$$\left(1-p\right)L_{\phi }\left[\hat{\phi }\left(\eta ,p\right)-\phi_{0}\left(\eta \right)\right]=p\hbar_{\phi }N_{\phi }\left[\hat{f}(\eta ,p),\hat{\theta }(\eta ,p),\hat{\phi }\left(\eta ,p\right)\right],$$ (21)

with

$$\begin{array}{l} {\hat{f}(\eta ;p)|}_{\eta =0}=0,\;{\frac{\partial \hat{f}(\eta ;p)}{\partial \eta }|}_{\eta =0}=1,\;{\frac{\partial \hat{f}(\eta ;p)}{\partial \eta }|}_{\eta =\infty }=0,\\ {\hat{\theta }(\eta ;p)|}_{\eta =0}=1,\;{\hat{\theta }(\eta ;p)|}_{\eta =\infty }=0,\\ {\hat{\phi }(\eta ;p)|}_{\eta =0}=1,\;{\hat{\phi }(\eta ;p)|}_{\eta =\infty }=0, \end{array}$$ (22)

where *p* ∈ [0,1] is the embedding parameter and ℏ_*f*_, ℏ_*θ*_ and ℏ_*ϕ*_ are the non-zero auxiliary parameters. Moreover the non-linear operators **N**_*f*_, **N**_*θ*_ and **N**_*ϕ*_ are given by

$$\begin{array}{l} N_{f}\left[\hat{f}(\eta ;p),\hat{\theta }(\eta ,p)\right]=\frac{\partial^{3}\hat{f}(\eta ,p)}{\partial \eta^{3}}+\hat{f}(\eta ,p)\frac{\partial^{2}\hat{f}(\eta ,p)}{\partial \eta^{2}}-{\left(\frac{\partial \hat{f}(\eta ,p)}{\partial \eta }\right)}^{2}\\ +\beta_{1}\left\{2\hat{f}(\eta ,p)\frac{\partial \hat{f}(\eta ,p)}{\partial \eta }\frac{\partial^{2}\hat{f}(\eta ,p)}{\partial \eta^{2}}-{\left(\hat{f}(\eta ,p)\right)}^{2}\frac{\partial^{3}\hat{f}(\eta ,p)}{\partial \eta^{3}}\right\}+G\hat{\theta }(\eta ,p)\cos\alpha \\ -\beta_{2}\left\{\begin{array}{c} {\left(\hat{f}(\eta ,p)\right)}^{3}\frac{\partial {\hat{f}}^{4}(\eta ,p)}{\partial \eta^{4}}-2\hat{f}(\eta ,p){\left(\frac{\partial \hat{f}(\eta ,p)}{\partial \eta }\right)}^{2}\frac{\partial^{2}\hat{f}(\eta ,p)}{\partial \eta^{2}}\\ -3{\left(\hat{f}(\eta ,p)\right)}^{2}\frac{\partial^{2}\hat{f}(\eta ,p)}{\partial \eta^{2}}\frac{\partial^{2}\hat{f}(\eta ,p)}{\partial \eta^{2}} \end{array}\right\}+\beta_{3}\left\{{\left(\frac{\partial^{2}\hat{f}(\eta ,p)}{\partial \eta^{2}}\right)}^{2}-\hat{f}(\eta ,p)\frac{\partial^{4}\hat{f}(\eta ,p)}{\partial \eta^{4}}\right\}\\ -M^{2}\left\{\frac{\partial \hat{f}(\eta ,p)}{\partial \eta }-\beta_{1}\left(\hat{f}(\eta ,p)\frac{\partial^{2}\hat{f}(\eta ,p)}{\partial \eta^{2}}\right)+\beta_{2}\left({\left(\hat{f}(\eta ,p)\right)}^{2}\frac{\partial^{3}\hat{f}(\eta ,p)}{\partial \eta^{3}}\right)\right\}, \end{array}$$ (23)

$$\begin{array}{l} N_{\theta }\left[\hat{f}(\eta ;p),\hat{\theta }(\eta ;p),\hat{\phi }(\eta ;p)\right]=\frac{\partial^{2}\hat{\theta }(\eta ;p)}{\partial \eta^{2}}+\mathrm{Pr}\left(\hat{f}(\eta ,p)\frac{\partial \hat{\theta }(\eta ;p)}{\partial \eta }-2\hat{\theta }(\eta ;p)\frac{\partial \hat{f}(\eta ,p)}{\partial \eta }\right)\\ +\mathrm{Pr}\left(N_{t}\frac{\partial^{2}\hat{\theta }(\eta ,p)}{\partial \eta^{2}}+N_{b}\frac{\partial \hat{\phi }(\eta ;p)}{\partial \eta }\frac{\partial \hat{\theta }(\eta ;p)}{\partial \eta }\right)+A\frac{\partial \hat{f}(\eta ;p)}{\partial \eta }+B\hat{\theta }(\eta ;p), \end{array}$$ (24)

$$N_{\phi }\left[\hat{f}(\eta ;p),\hat{\theta }(\eta ;p),\hat{\phi }(\eta ;p)\right]=\frac{\partial^{2}\hat{\phi }(\eta ;p)}{\partial \eta^{2}}+Sc\left(\begin{array}{l} \hat{f}(\eta ,p)\frac{\partial \hat{\phi }(\eta ;p)}{\partial \eta }\\ -2\hat{\phi }(\eta ;p)\frac{\partial \hat{f}(\eta ,p)}{\partial \eta } \end{array}\right)+\frac{N_{b}}{N_{t}}\frac{\partial^{2}\hat{\theta }(\eta ;p)}{\partial \eta^{2}}.$$ (25)

It is noted that for *p* = 0 and *p* = 1 we have

$$\begin{array}{l} \hat{f}(\eta ;0)=f_{0}(\eta ),\;\hat{f}(\eta ;1)=f(\eta ),\;\hat{\theta }(\eta ;0)=\theta_{0}(\eta ),\;\hat{\theta }(\eta ;1)=\theta (\eta ),\\ \hat{\phi }(\eta ;0)=\phi_{0}(\eta ),\;\hat{\phi }(\eta ;1)=\phi (\eta ), \end{array}$$ (26)

and making use of the Taylors’ series expansion we get

$$\hat{f}(\eta ;p)=f_{0}(\eta )+\overset{\infty }{\underset{m=1}{\sum }}f_{m}(\eta )p^{m},$$ (27)

$$\hat{\theta }(\eta ;p)=\theta_{0}(\eta )+\overset{\infty }{\underset{m=1}{\sum }}\theta_{m}(\eta )p^{m},$$ (28)

$$\hat{\phi }(\eta ;p)=\phi_{0}(\eta )+\overset{\infty }{\underset{m=1}{\sum }}\phi_{m}(\eta )p^{m},$$ (29)

where

$$f_{m}(\eta )={\frac{1}{m!}\frac{\partial^{m}f(\eta ;p)}{\partial p^{m}}|}_{p=0},\;\theta_{m}(\eta )={\frac{1}{m!}\frac{\partial^{m}\theta (\eta ;p)}{\partial p^{m}}|}_{p=0},\;\phi_{m}(\eta )={\frac{1}{m!}\frac{\partial^{m}\phi (\eta ;p)}{\partial p^{m}}|}_{p=0}.$$ (30)

The corresponding deformation problems at the *mth* order are

$$L_{f}\left[f_{m}\left(\eta \right)-\chi_{m}f_{m-1}\left(\eta \right)\right]=\hbar_{f}R_{m}^{f}\left(\eta \right),$$ (31)

$$L_{\theta }\left[\theta_{m}\left(\eta \right)-\chi_{m}\theta_{m-1}\left(\eta \right)\right]=\hbar_{\theta }R_{m}^{\theta }\left(\eta \right),$$ (32)

$$L_{\phi }\left[\phi_{m}\left(\eta \right)-\chi_{m}\phi_{m-1}\left(\eta \right)\right]=\hbar_{\phi }R_{m}^{\phi }\left(\eta \right),$$ (33)

and

$$\begin{array}{l} f_{m}(0)=0,\;f_{m}^{\prime }(0)=0,\;f_{m}^{\prime }(\infty )=0,\;\theta_{m}(0)=0,\;\theta_{m}(\infty )=0,\\ \phi_{m}(0)=0,\;\phi_{m}(\infty )=0. \end{array}$$ (34)

Moreover

$$\begin{array}{l} R_{m}^{f}\left(\eta \right)=f_{m-1}^{\prime \prime \prime }(\eta )-\sum_{k=0}^{m-1}f_{m-1-k}^{\prime }f_{k}^{\prime }+\sum_{k=0}^{m-1}f_{m-1-k}f_{k}^{{}^{\prime \prime }}+\beta_{1}\sum_{k=0}^{\begin{array}{l} m-1 \end{array}}\left\{2f_{m-1-k}\sum_{l=0}^{k}f_{k-l}^{\prime }f_{l}^{{}^{\prime \prime }}-f_{m-1-k}\sum_{l=0}^{k}f_{k-l}f_{l}^{{}^{{}^{\prime \prime \prime }}}\right\}\\ +\beta_{2}\sum_{k=0}^{m-1}\left\{\begin{array}{c} f_{m-1-k}\sum_{l=0}^{k}f_{k-l}\sum_{p=0}^{l}f_{l-p}f_{p}^{{}^{\prime \prime \prime \prime }}-2f_{m-1-k}\sum_{l=0}^{k}f_{k-l}^{{}^{\prime }}f_{l}^{{}^{\prime \prime }}\\ -3f_{m-1-k}\sum_{l=0}^{k}f_{k-l}f_{l}^{{}^{{}^{\prime \prime \prime \prime }}} \end{array}\right\}+\beta_{3}\left\{f_{m-1-k}^{\prime \prime }\sum_{k=0}^{m-1}f_{k}^{\prime \prime }-f_{m-1-k}\sum_{k=0}^{m-1}f_{k}^{{}^{\prime \prime \prime \prime }}\right\}\\ -M^{2}\left\{f_{m-1}^{\prime }(\eta )-\beta_{1}\left(\sum_{k=0}^{m-1}f_{m-1-k}f_{k}^{{}^{\prime \prime }}\right)+\beta_{2}\left(f_{m-1-k}\sum_{l=0}^{k}f_{k-l}f_{l}^{{}^{{}^{\prime \prime \prime }}}\right)\right\}+G\theta_{m-1}\cos\alpha , \end{array}$$ (35)

$$R_{m}^{\theta }\left(\eta \right)=\theta_{m-1}^{\prime \prime }+\mathrm{Pr}\sum_{k=0}^{m-1}\left[\begin{array}{l} f_{m-1-k}\theta_{k}^{\prime }-f_{m-1-k}^{\prime }\theta_{k}\\ +N_{b}\phi_{m-1-k}\theta_{k}+N_{t}\sum_{k=0}^{m-1}\theta_{m-1-k}^{{}^{\prime }}\theta_{k}^{{}^{\prime }} \end{array}\right]+Af_{m-1}^{\prime }+B\theta_{m-1}^{},$$ (36)

$$R_{m}^{\phi }\left(\eta \right)=\phi_{m-1}^{\prime \prime }+Sc\sum_{k=0}^{m-1}\left[f_{m-1-k}\phi_{k}^{\prime }-f_{m-1-k}^{\prime }\phi_{k}\right]+\frac{N_{b}}{N_{t}}\theta_{m-1}^{\prime \prime },$$ (37)

and

$$\chi_{m}=\{\begin{array}{c} 0,\;m\leq 1,\\ 1,\;m>1. \end{array}.$$ (38)

## Convergence Analysis

**Fig 1:**
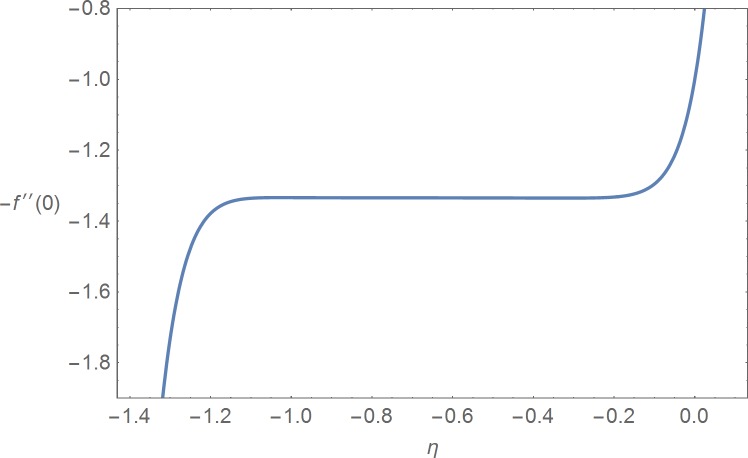
ℏ_*f*_-curve.

**Fig 2:**
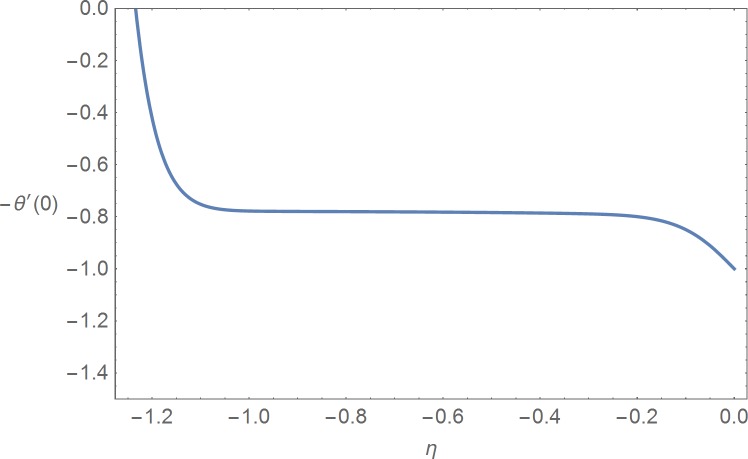
ℏ_*θ*_-curve.

**Fig 3:**
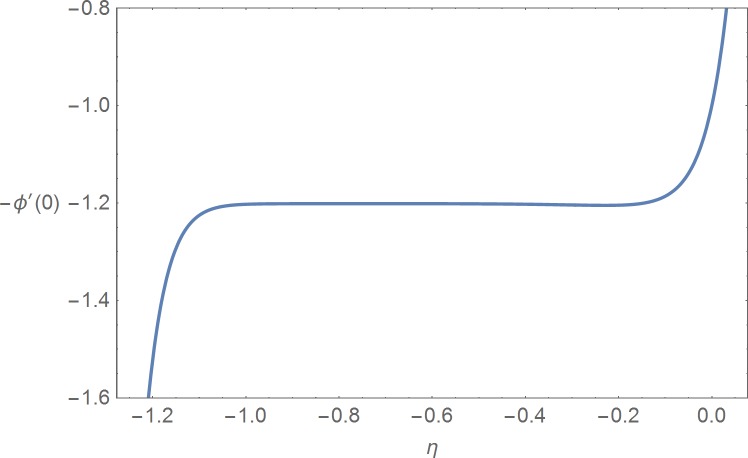
ℏ_*ϕ*_-curve.

It is noted that the nonlinear Eqs 19–21 contain the supporting parameters ℏ_*f*_, ℏ_*θ*_ and ℏ_*ϕ*_. These non-zero auxiliary parameters are very much useful in adjusting and controlling the convergence of the obtained solutions. In order to find the suitable domains we have plotted ℏ_*f*_-, ℏ_*θ*_- and ℏ_*ϕ*_-curves at 15^th^ order of approximation in the Figs 1–3 by selecting *β*_1_ = 0.2 = *β*_2_ = *β*_3_, *M* = 1.0 = Pr = *Sc*, *N*_*b*_ = 1.0, *N*_*t*_ = 1.0, *A* = 0.1 = *B*, *G* = 0.1 and *α* = *π*/4. These plots show that the admissible ranges are −1.2 ≤ ℏ_*f*_ ≤ −0.2, −1.15 ≤ ℏ_*θ*_ ≤ −0.25 and −1.15 ≤ ℏ_*ϕ*_ ≤ −0.25. Table 1 is prepared for the convergence of the series solutions. It is found that the convergence is achieved at 15^th^ order of approximations.

**Table 1:** Convergence of the derived solutions.

Order	Item 1	Item 2	Item 3
1	1.332251	0.906666	1.210000
2	1.333996	0.799134	1.201590
5	1.334381	0.789481	1.201401
10	1.334167	0.779619	1.201361
15	1.334161	0.779670	1.201322
20	1.334161	0.779670	1.201322
30	1.334161	0.779670	1.201322
40	1.334161	0.779670	1.201322

**Item 1: −*f*″(0)**, **Item 2: −*θ*′(0)**, **Item 3: −*ϕ*′(0)**

## Results and Discussion

**Fig 4:**
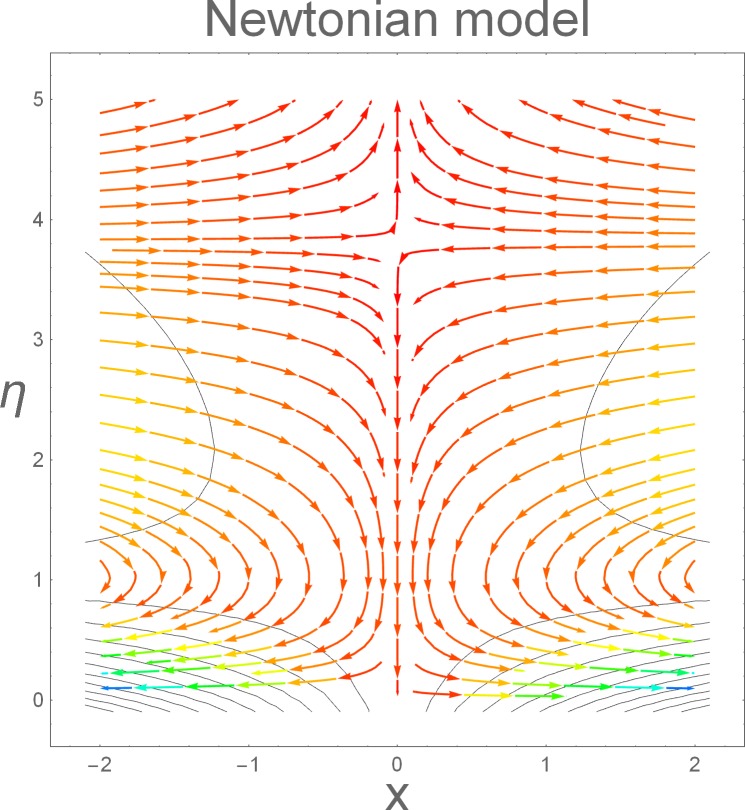
Streamlines for Newtonian model.

**Fig 5:**
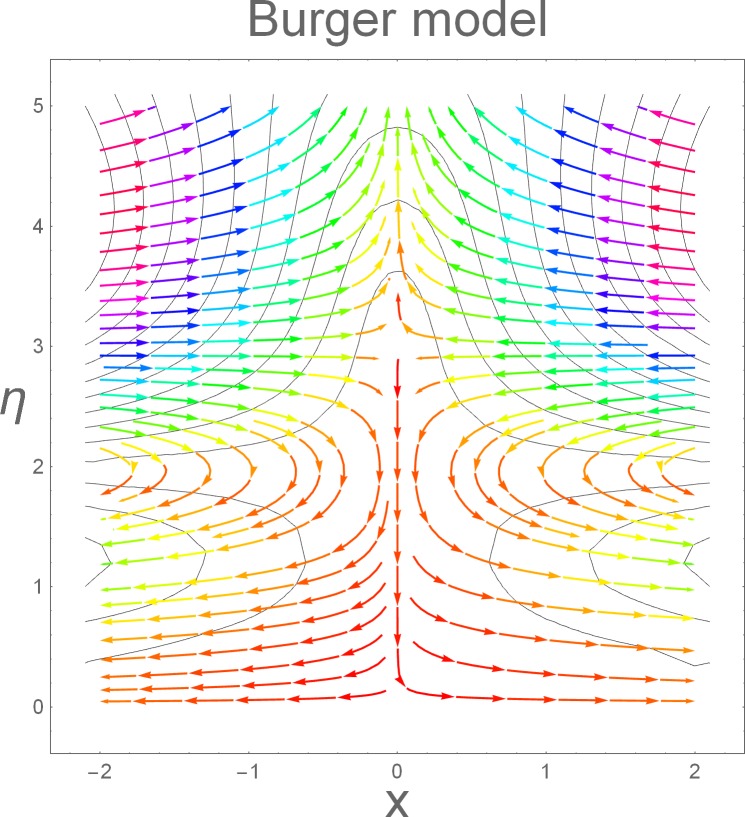
Streamlines for Burgers’ model.

**Fig 6:**
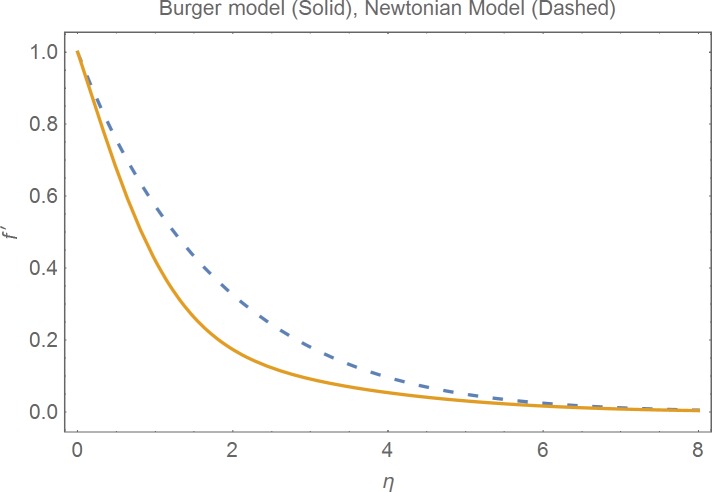
Comparison of Newtonian and Burger’s models.

**Fig 7:**
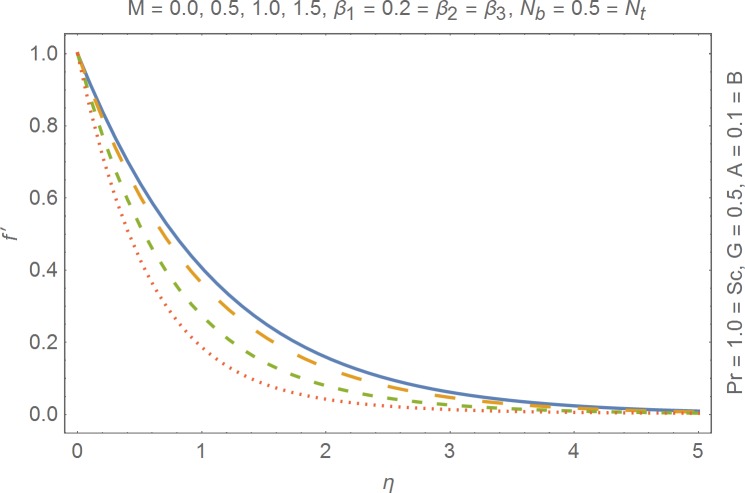
Influence of magnetic field on velocity profile.

**Fig 8:**
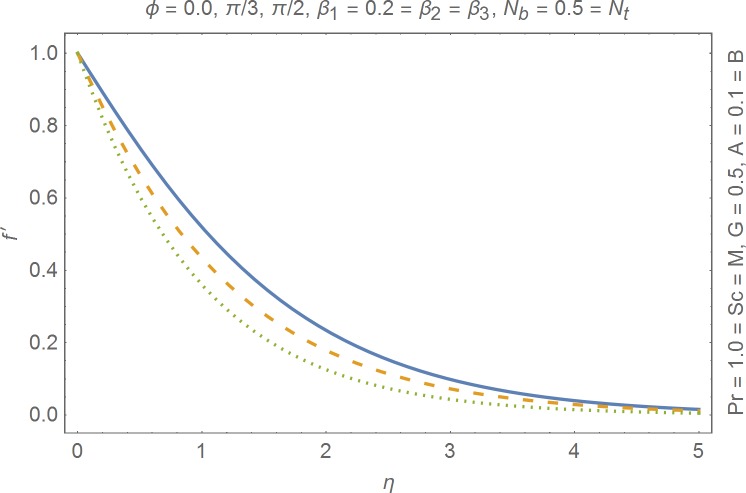
Influence of *ϕ* on velocity profile.

**Fig 9:**
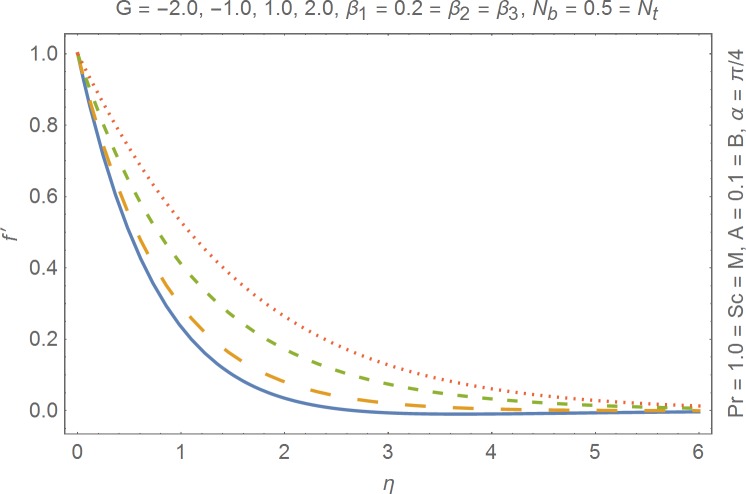
Influence of convection on velocity.

**Fig 10:**
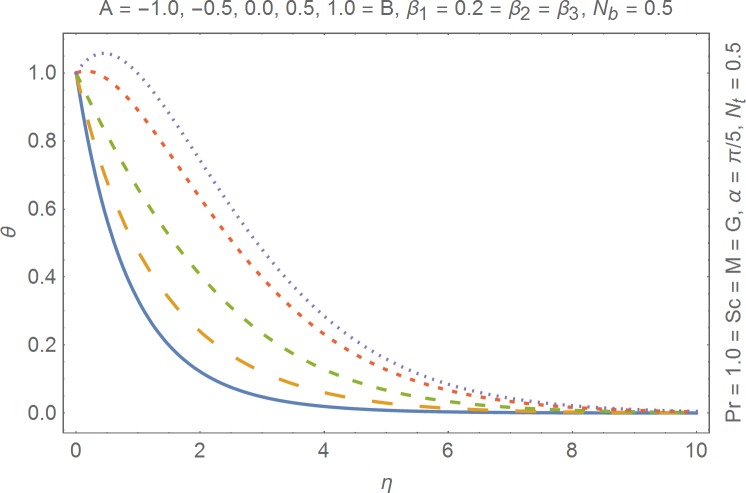
Influence of non-uniform heat generation/absorption on temperature.

**Fig 11:**
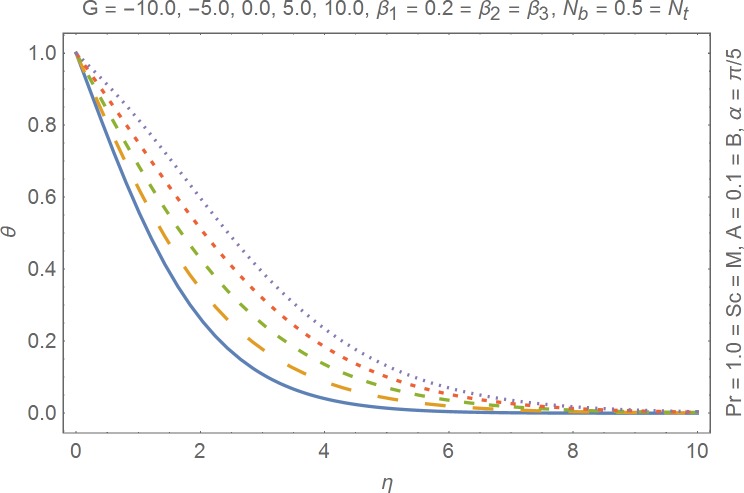
Influence of convection parameter on temperature.

**Fig 12:**
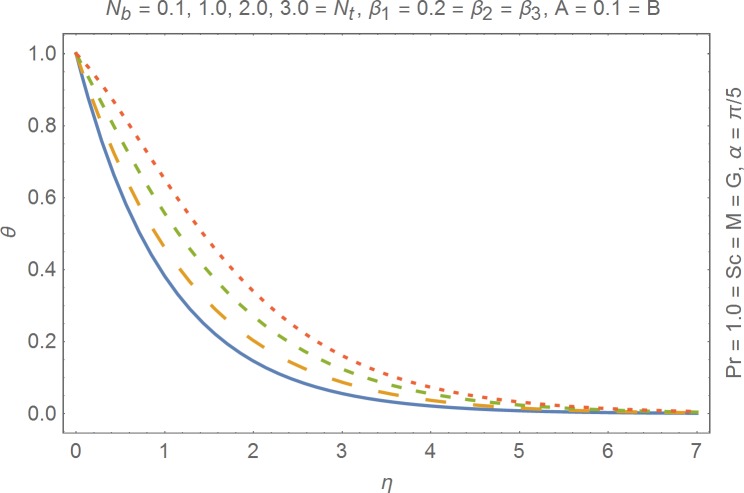
Influence of thermophoresis and Brownian motion on temperature.

**Fig 13:**
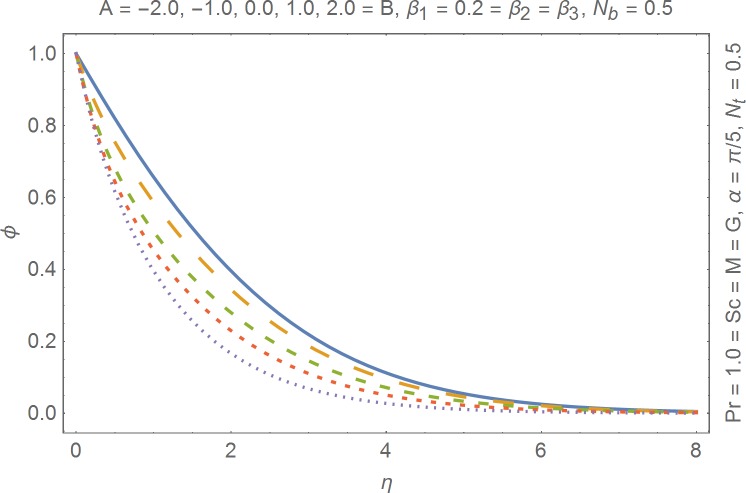
Influence of non-uniform heat generation/absorption on concentration.

**Fig 14:**
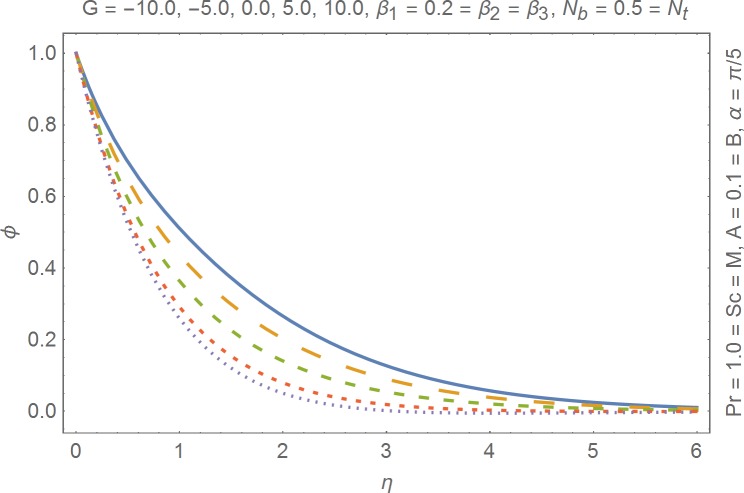
Influence of convection parameter on concentration.

**Fig 15:**
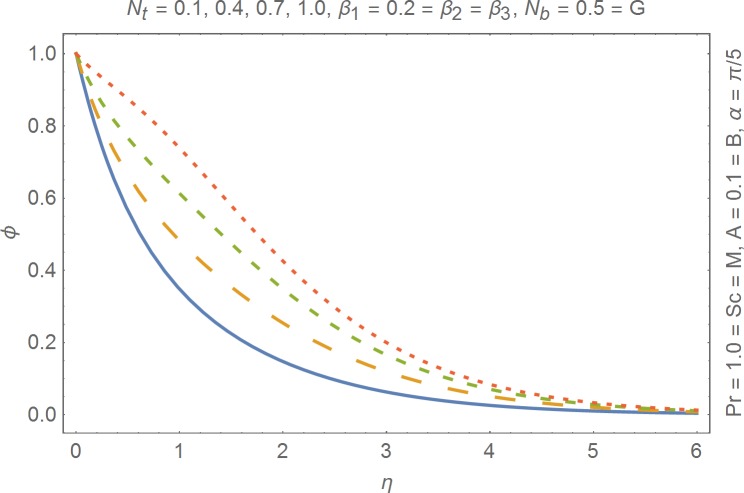
Influence of thermophoresis on concentration.

**Fig 16:**
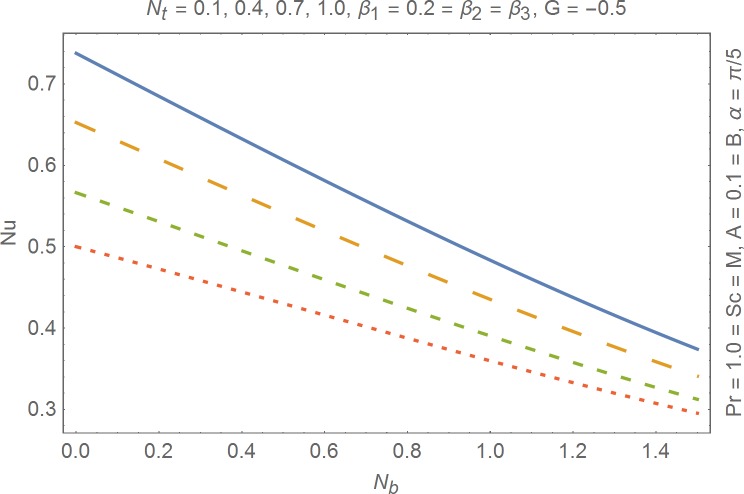
Influence of thermophoresis and Brownian motion on Nusselt number.

**Fig 17:**
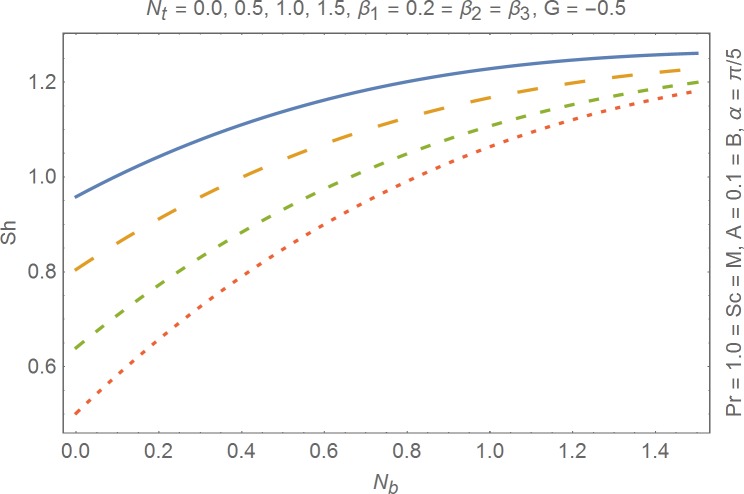
Influence of thermophoresis and Brownian motion on Sherwood number.

The aim of this section is to present the rheological and physical effects on the flow field. For this purpose we have prepared Figs 4–17. Figs 4 and 5 present the stream lines for the Newtonian and Burgers’ fluid models. It is observed that streamlines for Burgers’ fluid model show different rheology when compared with the streamlines for Newtonian fluid. The different is due to the addition relaxation and retardation effects which are present in the stress tensor for the Burgers’ fluid. Fig 6 presents a comparative study between Newtonian and Burgers’ fluid models. It is observed that velocity profile and momentum boundary layer are lessor for the Burgers’ fluid when compared with the Newtonian fluid model. From physical point of view we can say that addition rheological effects namely the Deborah numbers (*β*_1_, *β*_2_ and *β*_3_) are non-zero for Burgers’ fluid model. Nonzero values of *β*_1_, *β*_2_ and *β*_3_ correspond to viscous as well as elastic effects which retards the flow and hence the boundary layer will be thinner which is noted in Fig 6. The influence of magnetic field *M* on the flow field are presented in the Fig 7. It is observed that magnetic field retards the flow and thinner the momentum boundary layer. Physically it is noted that when any fluid is subjected to a magnetic field then the apparent viscosity increases. The outcome of which is that the fluid's ability to transmit force can be controlled with the help of an electromagnet which gives rise to its many possible control-based applications including MHD power generation, electromagnetic casting of metals, MHD ion propulsion etc. Fig 8 presents the effects of inclination angle on the velocity profile. It is noted that angle *α* varies from 0−*π*/2. Angle *α* = 0 represents the case of vertical wall whereas *α* = *π*/2 represents the horizontal wall. We noticed that when *α* varies from 0−*π*/2 the strength of buoyancy force decrease which results into decrease in velocity profile and momentum boundary layer. Fig 9 presents the influence of convection parameter *G* on the flow field. It is observed that velocity field is an increasing function of *G*. It is because of the fact that the larger values of *G* induces a strong buoyancy force which enhances the fluid velocity and increases the momentum boundary layer. The combined effects of internal heat generation/absorption parameters *A* and *B* on the temperature profile are presented in the Fig 10. It is noted that the temperature profile *θ*(*η*) decreases for internal heat generation/absorption phenomenon (i.e. *A < 0* and *B < 0*) whereas for internal heat generation/absorption effects (i.e. *A > 0* and *B > 0*) the temperature profile *θ*(*η*) increases. Fig 11 presents the effects of convection parameters *G* on the temperature profile *θ*(*η*). It is noted that *θ*(*η*) is an increasing function of *G*. Fig 12 presents the combined effects of thermophoresis and the Brwonian motion of the temperature profile *θ*(*η*). It is noted that *θ*(*η*) increases with an increase in thermophoresis and the Brownian motion. From physical point of view we can say that an increase in the strength of Brownian motion and thermophoresis cause an effective movement of the nanoparticles which enhances the thermal conductivity of the fluid which results into enhancement of the fluid temperature. Figs 13 and 14 portray the effects of non-uniform heat source/sink and convection phenomenon on the concentration profile *ϕ*(*η*). Both graphs show that the obtained results are quite opposite when compared with the effects on temperature profile as presented in Figs 9 and 10. The effects of nanofluid parameter *N*_*t*_ on the concentration profile are elucidated in the Fig 15. It is seen that the concentration profile *ϕ*(*η*) and the concentration boundary layer are increasing functions of *N*_*t*_. Figs 16 and 17 present the influence of thermophoresis and Brownian motion on the local Nusselt and Sherwood numbers. It is noted that the obtained results in both plots are qualitatively similar.

Table 2 presents a comparative study of the obtained results with those of Ref. [25]. It is noted that the present results in a limiting case are in a nice agreement with an already (Ref. [25]).

**Table 2:** Comparison of obtained results with Ref. [25] in a limiting case.

B	Pr	Our results	Ref. [25]
-1.0	1.0	-1.710936	-1.710937
-2.0	2.0	-2.486000	-2.458997
-3.0	3.0	-3.028170	-3.028177
-4.0	4.0	-3.585189	-3.585192
-5.0	5.0	-4.028533	-4.028540

## Concluding Remarks

In this paper, an analysis is presented for the two-dimensional boundary layer flow of Burgers’ nanofluid over an inclined wall with non-uniform heat source/sink. The important observations are as follows:

- Streamlines for Newtonian and Burger model are prepared which show the difference of rheology.
- Velocity field and momentum boundary layer are lessor for Burger model due to extra viscoelastic effects.
- Larger values of magnetic field retards the fluid flow.
- Non-uniform heat generation and absorption phenomenon has opposite effects respectively.
- Thermophoresis and Brownian motion enhance the molecular movement which increases the fluids’ temperature and concentration.
- Results for assisting and opposing flow situations are quite opposite.

## References

[1] Jamil M and Fetecau C (2010) Some exact solutions for rotating flows of a generalized Burgers’ fluid in cylindrical domain, J. Non-Newtonian Fluid, 165: 1700–1712.

[2] Fetecau C, Hayat T, Khan M and Fetecau C (2010) A note on longitudinal oscillations of a generalized Burger fluid in cylindrical domains, J. Non-Newtonan Fluid, 165: 350–361

[3] Xue C, Nie J and Tan W C (2008) An exact solution of start-up flow for the fractional generalized Burgers’ fluid in a porous half space, Nonlinear Analysis: Theo. Math. Appl., 69: 2086–2094.

[4] Khan M, Anjum A, Fetecau C and Qi H (2010) Exact solutions for some oscillating motion of a motions of a fractional Burgers’ fluid, Math. Comp. Mod., 51: 682–692.

[5] Liu Y, Zheng L and Zhang X (2011) MHD flow and heat transfer of a generalized Burgers’ fluid due to an exponential accelerating plate with the effects of radiation, Comp. Math. Appl., 62: 3123–3131.

[6] Javed M, Hayat T and Alsaedi A (2014) Peristaltic flow of Burgers’ fluid with complaint walls and heat transfer, Appl. Math. Comp, 244: 654–671.

[7] Awais M, Hayat T and Alsaedi A (2015) Investigation of heat transfer in flow of Burgers’ fluid during a melting process, J. Egyp. Math. Soc., 23: 410–415.

[8] Choi S U S (1995) Enhancing thermal conductivity of fluids with nanoparticles. ASME Int. Mech. Engng., 66: 99–105.

[9] Khan W I and Pop I (2010) Boundary-layer flow of a nanofluid past a stretching sheet. Int. J. Heat Mass Transfer, 53: 2477–2483.

[10] Makinde O D and Aziz A (2011) Boundary layer flow of a nanofluid past a stretching sheet with a convective boundary condition. Int. J. Therm. Sci., 50: 1326–1332.

[11] Rana P and Bhargava R (2012) Flow and heat transfer of a nanofluid over a nonlinearly stretching sheet: A numerical study, Com. Nonlinear Sci. Numer. Simulat., 17: 212–226.

[12] Hamad M A A and Ferdows M (2012) Similarity solution of boundary layer stagnation-point flow towards a heated porous stretching sheet saturated with a nanofluid with heat absorption/generation and suction/blowing: A Lie group analysis, Com. Nonlinear Sci. Numer. Simulat., 17: 132–140.

[13] Awais M, Hayat T, Irum S and Alsaedi A (2015) Heat generation/absorption effects in a boundary layer stretched flow of Maxwell nanofluid: Analytic and numeric solutions, PLOS ONE, 10: e0129814 doi: 10.1371/journal.pone.0129814 26115101 10.1371/journal.pone.0129814 PMC4482663

[14] Liao S J (2009) Notes on the homotopy analysis method: Some definitions and theorems, Commun. Nonlinear. Sci. Numer. Simulat. 14: 983–997.

[15] Rashidi M M, Pour S A M, Hayat T and Obaidat S (2012) Analytic approximate solutions for steady flow over a rotating disk in porous medium with heat transfer by homotopy analysis method, Comp. Fluids, 54: 1–9.

[16] Rashidi M M, Feridoonimehr N, Hosseini A, Beg O A and Hung T K (2014) Homotpy simulations of nanofluid dynamics from a non-linearly stretching isothermal permeable sheet with transpiration, Meccanica, 49: 469–482.

[17] Abbasbandy S (2011) Approximate analytical solutions to thermo-poroelactic equations by means of the iterated homotopy analysis method, Int. J. Comp. Math., 88: 1763–1775.

[18] Abbasbandy S and Jalili M (2013) Determination of optimal convergence control parameter value in homotopy analysis method, Numerical Agorithms, 64: 593–605.

[19] Awais M, Hayat T, Nawaz M and Alsaedi A (2015) Newtonian heating, thermal-diffusion and diffusion-thermo effects in an axisymmetric flow of a Jeffery fluid over a stretching surface, Braz. J. Chem. Engng., 32: 555–561.

[20] Hayat T, Hussain Z, Farooq M and Alsaedi A (2016) Effects of homogeneous and heterogeneous reactions and melting heat in the viscoelastic fluid flow, J. Mol. Liq., 215: 749–755.

[21] Khan M and Malik R (2016) Forced convective heat transfer to Sisko nanofluid past a stretching cylinder in the presence of variable thermal conductivity, J. Mol. Liquids, 218: 1–7.

[22] Tsai R, Huang K H, Huang J S (2008) Flow and heat transfer over an unsteady stretching surface with non-uniform heat source, Int. Comm. Heat Mass Transfer, 35: 1340–1343.

[23] Rashidi M M and Hassan H (2014) An analytic solution of micropolar flow in a porous channel with mass injection using homotopy analysis method, Int. J. Numer. Meth. Heat Fluid Flow 24: 419–437.

[24] Rashidi M M, Shooshtari A, Beg O A (2012) Homotopy Perturbation Study of Nonlinear Vibration of Von Karman Rectangular Plates, Comp. Stru. 106–107: 46–55.

[25] Rashidi M M, Momoniat E and Rostami B (2012), Analytic approximate solutions for MHD boundary-layer viscoelastic fluid flow over continuously moving stretching surface by homotopy analysis method with two auxiliary parameters, J. Applied Math., 2012: 780415.

